# Supplementary material for: Passive margins in accreting Archaean archipelagos signal continental stability promoting early atmospheric oxygen rise
Source: Nat Commun. 2022 Dec 19;13:7821. doi: 10.1038/s41467-022-35559-w (PMC9763395; doi:10.1038/s41467-022-35559-w)
Supplement: Supplementary file 1 — Supplementary Information [file 41467_2022_35559_MOESM1_ESM.pdf]

Supplementary Information

Supplementary Discussion 1

Methodology Applied in Chronology Study

Analytical methods

Analyses of 19ZK-0, -2, -5, -6 were conducted at the John de Laeter Centre, Curtin University, using a 30 μm diameter laser spot (following two cleaning pulses) with a 30s ablation time, 4 Hz laser repetition rate and on-sample laser energy of 2.0 J cm^{-2}. Ultrahigh purity He (350 mL/min) and N_2 (3.8 mL/min) was used to flush the cell and high purity Ar was employed as the plasma carrier gas (flow rate 0.98 L/min). Isotopic intensities were measured on an Agilent 8900 triplequadrupole (QQQ) ICP-MS, whose $^{206}Pb/^{238}U$ ages calculated for all zircon age standards, treated as unknowns, were found to be within 3% of the accepted value. The time-resolved mass spectra were reduced using the U_Pb_Geochronology4 data reduction scheme in Iolite3.5[1]. Analyses of 19ZK-4, -6.1, -7, 19YY-2, 21YY-3, 20NQ-1.2, -1.3, -3.2, -3.3 were conducted at the Wuhan SampleSolution Analytical Technology Co., Ltd., Wuhan, China. Detailed operating conditions for the laser ablation system and the ICP-MS instrument and data reduction are the same as description by[2]. Laser sampling was performed using a GeolasPro laser ablation system that consists of a COMPexPro 102 ArF excimer laser (wavelength of 193 nm and maximum energy of 200 mJ) and a MicroLas optical system. An Agilent 7700e ICP-MS instrument was used to acquire ion-signal intensities. 21YY-9 and 19YY-2.1 were analyzed at the State Key Laboratory of Geological Processes and Mineral Resources, China University of Geosciences, Wuhan. Experiments were performed on an Agilent 7900 ICP-MS instrument (Agilent Technology, Tokyo, Japan) in combination with an ArF excimer laser (λ = 193 nm) (Geolas HD, MicroLas Göttingen, Germany). All analyses were performed with a laser spot size of 32 μm, a repetition rate of 5 Hz and a fluence of 8 J cm^{-2} in this study. International glass standard NIST 610 was used as the primary standard to calculate elemental concentrations and to correct for instrument drift. The primary age reference material used in this study was GJ-1 (599.8 ± 1.7 Ma[3]) with 91500 (1062.4 ± 0.4 Ma[4]) and Tanz (566.16 ± 0.77 Ma[5]) as secondary age standards. Off-line selection and integration of background and analytical signals, and time-drift correction and quantitative calibration for trace element analyses and U-Pb dating were performed by ICPMSDataCal[6]. Dates where

the concordant values > 105% or < 95% were considered to be excessively discordant and were not considered in the detrital age calculations.

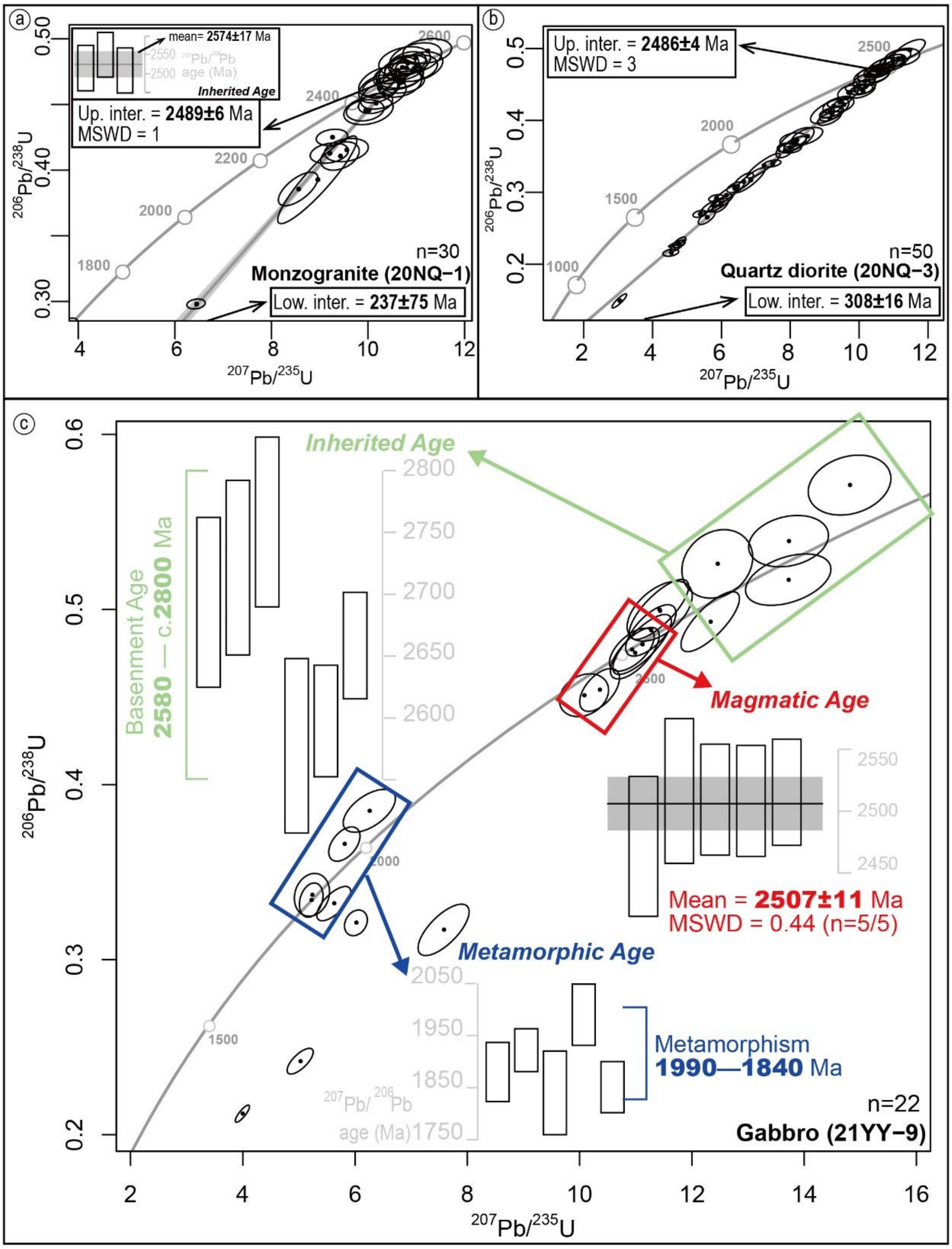

Supplementary Figure 1. Concordia diagram of zircon ages from intrusions (data from Supplementary Dataset 1). (a): 20NQ-1, monzogranite intruding basement. (b): 20NQ-3, quartz diorite intruding basements. (c): 21YY-9, deformed gabbro intruding Unit 2.

MDA Calculations

The YSG is calculated by sorting each age plus 2σ uncertainty and selecting the first analysis in this list[7]. As defined this way, an older/more precise analysis may be selected instead of a younger/less precise analysis[8]. Youngest Cluster Overlapping at 1σ or 2σ are calculated as the weighted mean ages of the youngest cluster that overlaps at 1σ and 2σ respectively, where the cluster is defined by the age minus the uncertainty of the oldest grain is less than the sum of the youngest grain's age and uncertainty. Both YPP and YC ages are calculated via the Python Based detritalpy program[9]. The YSP age is the weighted mean of the youngest group of two or more analyses that have a MSWD that is closest to one, which is calculated manually following the procedure proposed by[10].

Supplementary Discussion 2

Zircon Geochronology Data Description

Zircon Textures Under CL

21YY-3 (Unit 2 basal sandstone) zircons are subrounded, poorly sorted, coarse-grained, and fragmented. Zircons with cores of oscillatory zoning indicate an igneous provenance, and the bright metamorphic rims, broken grains, and semi-rounded grain edges confirms the sedimentary genesis (Supplementary Fig. 2a), probably with limited transportation distance based on the amount of rounding. The largest grains can reach 200 μm, while the smallest ones are merely 20–50 μm.

19ZK-567 (Unit 2 higher sandstone) zircons are rounded to subrounded fragments. Cores of zircons show all types of zoning from dense oscillatory, generated in felsic igneous rock, to wide zoning, from mafic magma, which implies a complex combination of provenance (Supplementary Fig. 2b) with mafic and felsic magmatic rocks. The edges of these oval-shaped zircon grains often truncate the internal oscillatory zoning, suggesting a moderate to high degree of weathering or long-distance transport. The grains have lengths of ~ 40–120 μm and widths of ~ 40–80 μm.

19ZK-024 (Unit 3 sandstone) zircons are mostly subhedral and subrounded-subprismatic, poor-shorted, and broken into fragments. Although dense oscillatory zoning is well-developed, there are zircons with thin metamorphic rims over fragments, indicating a post erosion-deposition metamorphic event, which is a typical index of sedimentary genesis (Supplementary Fig. 2c). The subhedral morphology could imply short distance and duration from erosion to deposition. The zircon in this unit is less fragmented than 19ZK-567, indicating a nearby provenance. The sizes of zircon grains vary greatly, from the largest being about 200 μm to the smallest being less than 50 μm.

19YY-2 (Unit 3 metapelite in marble-metapelite) zircons are very fine-grained, well-sorted and rounded (Supplementary Fig. 2d), consistent with the host metapelite (mudstone). Detritus shows dense oscillatory zoning, and relatively thin metamorphic rims. As most grains are around 30–40 μm, similar to the size of the laser spots, leading to strong influence of metamorphic contamination during U-Pb dating.

21YY-9 (gabbro sill) (Supplementary Fig. 2e) are composed of zoned magmatic zircons and zircons with sector textures due to later metamorphic growth. Zircons with igneous cores can be further divided into those with core textures that fit the morphology, and those that do not match the external morphology. The former

represent zircon grown in the magma, recording magmatic intrusion, while the latter texture could indicate preexisting zircon detritus in the sedimentary rock, captured by the mafic dyke during intrusion. These inherited grains may therefore contain xenocrystic cores that record the timing of magma passage through a “hidden” older basements.

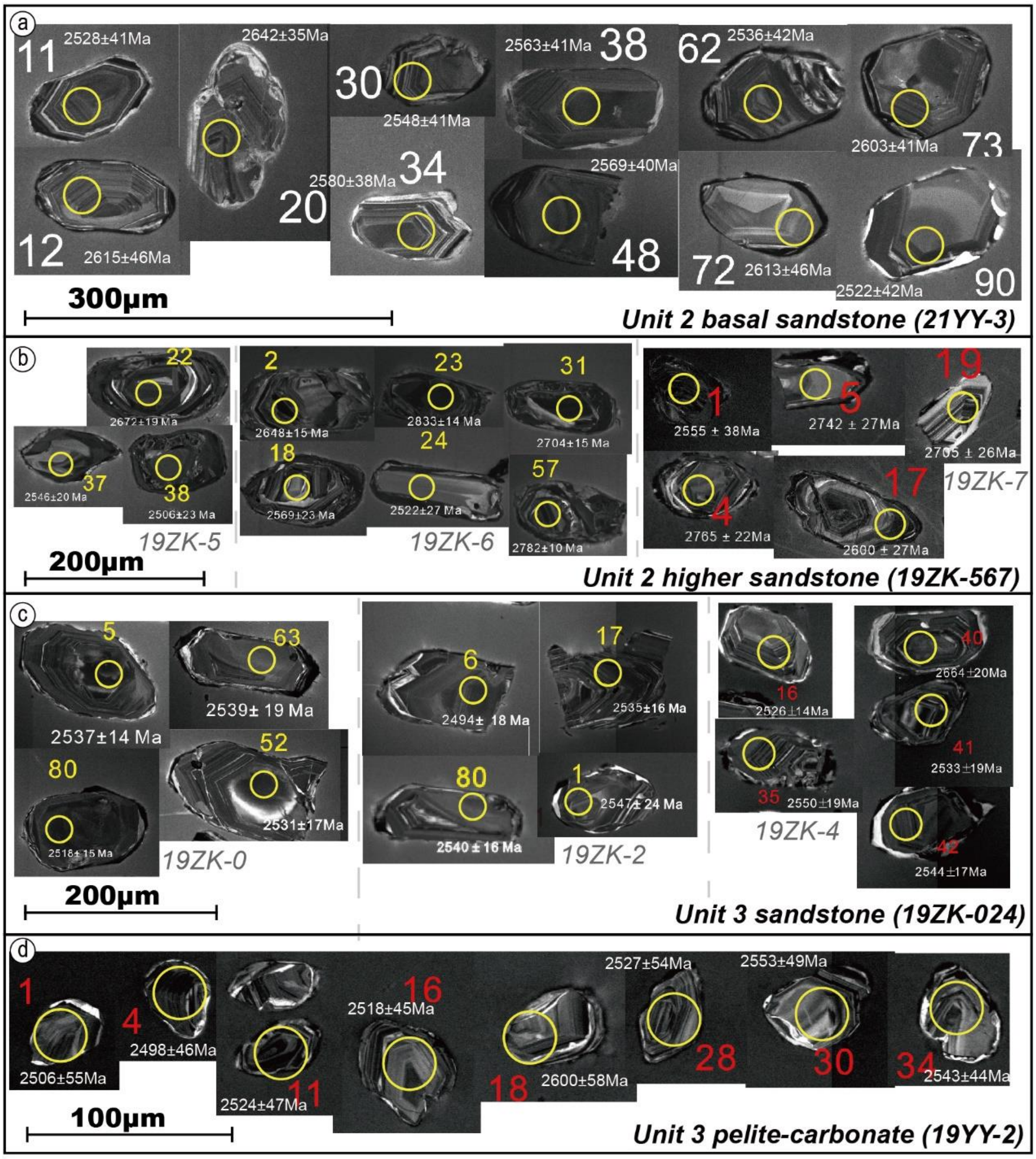

Supplementary Figure 2. Zircon image under CL (continued in next page). (a): 21YY-3, (meta)sandstone in Unit 2. (b): 19ZK-567, (meta)graywacke in Unit 2. (c): 19ZK-024, quartzofeldspathic (meta)arenite in Unit 3. (d): 19YY-2, metapelite in Unit 3. (e) 21YY-9, gabbro intruding Unit 2. (f) 20NQ-1, monzogranite intruding basement. (g) 20NQ-3, quartz diorite.

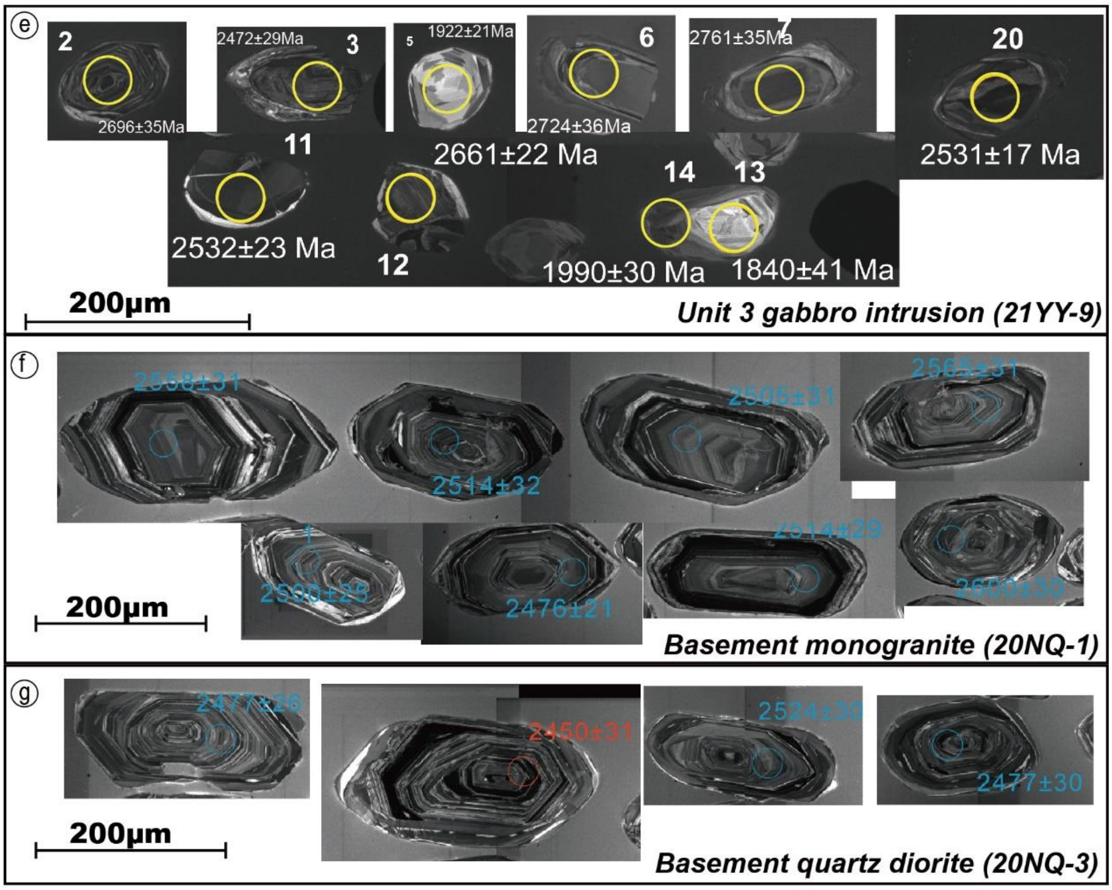

20NQ-1 (basement monzongranite) (Supplementary Fig. 2f) and 20NQ-3-(basement quartz diorite) (Supplementary Fig. 2g) are composed of 100–200 μm zircons with well-developed oscillatory zoning.

U-Pb Geochronology

$^{207}Pb/^{206}Pb$ age is more accurate for grains older than 1000 Ma[11]. In detrital zircon analysis we filtered out grains with age concordance < 95% to ensure the validity of the result for the detrital samples, but filtering was not performed on zircons from the intrusion.

21YY-3 (Unit 2 basal sandstone) zircons have U contents of 85–984 ppm, and Th/U of 0.311–1.171. Its Th/U > 0.1 showing that the analyzed spots are magmatic. $^{207}Pb/^{206}Pb$ ages are distributed from 2506±41 Ma to 2666±42Ma, with a 2528 Ma peak recognized (Fig. 8a).

19ZK-567 (Unit 2 higher sandstone) zircons have a wide range of U contents of (14–1228 ppm), and Th/U of 0.13–2.35. The Th/U > 0.1 shows that the analyzed spots were placed on magmatic grains, and $^{207}Pb/^{206}Pb$ ages are distributed from 2498±17 Ma

to 2833±14Ma. There is a strong concentration of ages around a peak at 2522 Ma, and gentle peaks noted at 2682 Ma and 2734 Ma (Fig. 8b). The wider range of elemental content and age distribution suggest mixing of source material.

19ZK-024 (Unit 3 sandstone) zircons have U contents of 39–1345 ppm and Th/U ratios of 0.51–1.88, interpreted as detrital grains derived from igneous rocks. $^{207}Pb/^{206}Pb$ ages are distributed from 2496 ± 15 Ma to 2555±28 Ma, with one analysis at 2658 ± 29 Ma. There is an intense young peak at 2517 Ma (Fig. 8c), indicating major input of young material.

19YY-2 (Unit 3 marble-metapelite) zircons have U contents of 168–859 ppm and a Th/U ratio of 0.450–1.812, interpreted as detrital grains derived from igneous rock. $^{207}Pb/^{206}Pb$ age distributed from 2470±44 Ma to 2646±18 Ma, and an intense young peak at 2516 Ma (Fig. 8d).

21YY-9 (gabbro intrusion) yielded three groups of zircons for analysis: inherited, magmatic, and metamorphic. Apart from five analyses with concordance less than 95% that were discarded, six inherited zircons have a low U content (325–506 ppm) and igneous Th/U (0.372–0.747). Their $^{207}Pb/^{206}Pb$ ages are distributed from 2761±35 Ma to 2580±36 Ma (Supplementary Fig. 1a), significantly older than the magmatic events that formed this gabbro intrusion. Five grains were recognized as magmatic zircon in CL images. These have a wide range of U contents (191–1011 ppm) and moderate Th/U ratios (0.578–769), and their $^{207}Pb/^{206}Pb$ ages yielded a weighted mean of 2507±11 Ma (MSWD=0.44). Two analyzed spots overlapped the older inherited core and magmatic mantle, with ages around 2530 Ma. These are interpreted to represent mixing of the older basement age and the magmatic age. Five metamorphic zircons are defined by apparent sector zoning in CL images, and low Th/U ratio (0.004–0.014), which are typical signatures of metamorphic zircon. One zircon, with an outer mantle yielding 1990±30 Ma and inner core yielding 1840±41 Ma (Supplementary Fig. 1b) with little overlap, indicates continuous metamorphism from 1990 Ma to 1840 Ma related to a high-grade metamorphic event that reached high enough P-T conditions to totally reset the U-Pb systematics in these grains.

21NQ-1 (basement monzongranite) zircons generally yield magmatic ages, except for three outliers with significantly older ages. The majority of zircons have low to moderate U contents (90–488 ppm) and igneous Th/U ratios (0.246–1.134). Discordant data all falls on the discordant line with an upper intercept at 2489±6 Ma, interpreted as the magmatic age, with a lower intercept at 237±75 Ma interpreted as a

later alteration event age. The geological background of this later event remains unconstrained in this study, but is similar in age to widespread granite emplacement, uplift of extensional core complexes, the Taihang Mountains, and mineralization related to Mesozoic decratonization[8, 9, 10, 11]. The three exceptions are dated at 2565±31 Ma, 2600±30 Ma, and 2559±30 Ma, with concordance of 97%, 97% and 99% respectively, and they have low U contents (107–144 ppm) and igneous Th/U ratios (0.255 – 0.717). These three grains yielded a weighted mean age of 2574±17 Ma (Supplementary Fig. 1b), indicating earlier magmatic events from intruded country rock.

21NQ-3 (basement quartz diorite) are all late collision-related magmatic zircons. For those with concordance >95%, the U content is generally low to moderate (119–365 ppm), with one exception (1055 ppm), and their Th/U ratios all show igneous features (0.210–1.003). Together, they fall on a discordance line with an upper intercept of 2486±4 Ma and a lower intercept of 308±16 Ma. The latter age coincides with that in 20NQ-1 and could similarly be related to the Mesozoic decratonization and initial uplift of the Taihang ranges [12, 13, 14, 15].

Supplementary References

1. Paton C, Hellstrom J, Paul B, Woodhead J, Hergt J. Iolite: Freeware for the visualisation and processing of mass spectrometric data. *Journal of Analytical Atomic Spectrometry* **26**, 2508-2518 (2011).

2. Zong K, *et al.* The assembly of Rodinia: The correlation of early Neoproterozoic (ca. 900 Ma) high-grade metamorphism and continental arc formation in the southern Beishan Orogen, southern Central Asian Orogenic Belt (CAOB). *Precambrian Research* **290**, 32-48 (2017).

3. Jackson SE, Pearson NJ, Griffin WL, Belousova EA. The application of laser ablation-inductively coupled plasma-mass spectrometry to in situ U–Pb zircon geochronology. *Chemical Geology* **211**, 47-69 (2004).

4. Wiedenbeck M, *et al.* Three natural zircon standards for U-Th-Pb, Lu-Hf, trace

element and REE analyses. *Geostandards Newsletter* **19**, 1-23 (1995).

5. Hu Z*, et al.* Tanz zircon megacrysts: a new zircon reference material for the microbeam determination of U–Pb ages and Zr–O isotopes. *Journal of Analytical Atomic Spectrometry* **36**, 2715-2734 (2021).

6. Liu Y*, et al.* Reappraisement and refinement of zircon U-Pb isotope and trace element analyses by LA-ICP-MS. *Chinese Science Bulletin* **55**, 1535-1546 (2010).

7. Dickinson WR, Gehrels GE. Use of U–Pb ages of detrital zircons to infer maximum depositional ages of strata: A test against a Colorado Plateau Mesozoic database. *Earth and Planetary Science Letters* **288**, 115-125 (2009).

8. Sharman GR, Malkowski MA. Needles in a haystack: Detrital zircon U Pb ages and the maximum depositional age of modern global sediment. *Earth-Science Reviews* **203**, (2020).

9. Sharman G, Sharman J, Sylvester Z. detritalPy–A Python-based toolset for visualizing and analyzing detrital geo-thermochronologic data: The Depositional Record, v. 4. (2018).

10. Coutts DS, Matthews WA, Hubbard SM. Assessment of widely used methods to derive depositional ages from detrital zircon populations. *Geoscience Frontiers* **10**, 1421-1435 (2019).

11. Compston W, Williams I, Kirschvink J, Zichao Z, Guogan M. Zircon U-Pb ages for the Early Cambrian time-scale. *Journal of the Geological Society* **149**, 171-

184 (1992).

12. Kusky TM, Windley B, Zhai M-G. Tectonic evolution of the North China Block: from orogen to craton to orogen. *Geological Society, London, Special Publications* **280**, 1-34 (2007).

13. Zhu R-X, Yang J-H, Wu F-Y. Timing of destruction of the North China Craton. *Lithos* **149**, 51-60 (2012).

14. Zhang S-H, Zhao Y, Davis GA, Ye H, Wu F. Temporal and spatial variations of Mesozoic magmatism and deformation in the North China Craton: Implications for lithospheric thinning and decratonization. *Earth-Science Reviews* **131**, 49-87 (2014).

15. Wang Z, Kusky TM, Capitanio FA. Water transportation ability of flat-lying slabs in the mantle transition zone and implications for craton destruction. *Tectonophysics* **723**, 95-106 (2018).
