# Supplementary material for: Passive margins in accreting Archaean archipelagos signal continental stability promoting early atmospheric oxygen rise
Source: Nat Commun. 2022 Dec 19;13:7821. doi: 10.1038/s41467-022-35559-w (PMC9763395; doi:10.1038/s41467-022-35559-w)
Supplement: Supplementary file 3 — Description of Additional Supplementary Files [file 41467_2022_35559_MOESM3_ESM.pdf]

Description of Additional Supplementary Files:

Supplementary Dataset 1: LA-ICP-MS analysis result of zircon U-Pb analysis result from intrusions, with no data filtered.

Supplementary Dataset 2: LA-ICP-MS analysis result of detrital zircon U-Pb analysis result from metasediments, with data filtering those of
